# Dr. Harriet Noble Watson

**Published:** 1917-06

**Authors:** 


**Dr. Harriet Noble Watson,** N. Y. City Med. College for Women 1878, died at her home in Albion, May 4, aged 81. She had been a practitioner of Albion for 39 years. When the Western House of Refuge was founded in 1893, she was appointed attending physician and served 14 years.

Of 66 deaths of physicians listed in the Jour. of the A. M. A., April 28, 3 were suicides, 5 due to automobile accidents, 1 due to drowning at night while making a professional call, and one, a woman, was burned to death.

